# Mosaicism trisomy 10 in a 14-month-old child with additional neurological abnormalities: case report and literature review

**DOI:** 10.1186/s12887-018-1237-1

**Published:** 2018-08-06

**Authors:** Yang Gao, Yu-cong Ma, Yang-hua Ju, Ya-nan Li

**Affiliations:** 1 Department of Pediatrics, The First Hospital of Jilin University, Changchun, Jilin 130021 People’s Republic of China; 2 Department of Molecular Biology, Basic Medical College of Jilin University, Changchun, 130021 People’s Republic of China

**Keywords:** Mosaicism trisomy 10, Nervous system, Dandy–Walker syndrome, Patent ductus arteriosus

## Abstract

**Background:**

Trisomy 10 is very rarely diagnosed, especially in living persons. Most reports of trisomy 10 pertain to prenatal diagnosis of trisomy 10 in the fetus. In addition, trisomy 10 has been reported as part of partial chromosomal abnormalities in some leukemic cells and tumor specimens. Only 6 cases of mosaicism trisomy 10 have been reported so far. None of these reports pertain to living children with neurological abnormalities.

**Case presentation:**

We report the case of a 14-month-old girl who was brought for treatment of unusual facies, growth retardation, and patent ductus arteriosus. Karyotype analysis revealed a 47, XX, + 10/46, XX pattern. MRI showed characteristics of Dandy–Walker syndrome and ventricular enlargement in the brain.

**Conclusions:**

This case is distinguished by its extreme rarity and its potential for use as a reference case of this condition in clinical settings.

## Background

Trisomy 10 is very rarely diagnosed in live-born children; to our knowledge, there have only been 7 reported births of children with trisomy 10 [1]. Of these 7 cases, only 1 prenatal case (reported in 2001) was adjudged to be a complete Trisomy 10; however, at 35 weeks + 4 days of gestation, the mother underwent preterm labor with resultant intrauterine fetal death; the fetus (birth weight 1020 g) was delivered vaginally [2]. Case reports of the other 6 cases described live births with trisomy 10 mosaicism, with typical clinical features including feeding problems, growth retardation, failure to thrive, blepharophimosis, low-set ears, high-arched palate, retrognathia, long slender trunk, marked plantar/palmar furrows, cardiopathy, and early death. However, none of these case reports presented any evidence of manifestations in a live case or of complete trisomy 10 in all cells. Moreover, none of the previous cases had neurological involvement. In this report, we describe a case of mosaicism trisomy 10 that presented with neurological abnormalities.

## Case presentation

**Fig. 1:**
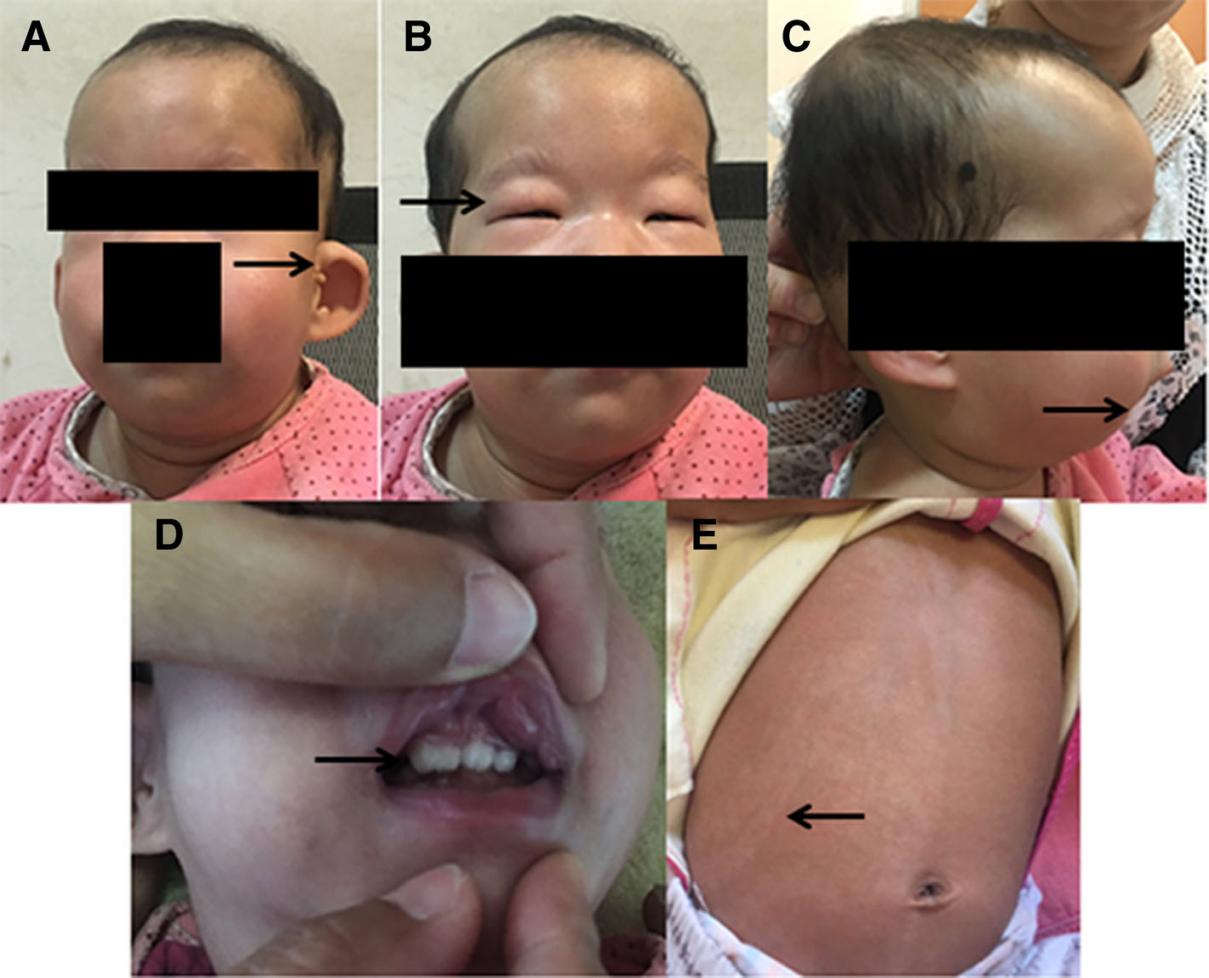
Photographs of the 14-month-old child with trisomy 10 showing morphological features: Front view (**a**): low-set ears and preauricular skin tag on the left side and (**b**) small palpebral fissure. Profile view (**c**): retrognathia. Oral examination (**d**): fused teeth. Skin (**e**): uneven color distribution of the skin and patchy pigmentation

A 14-month-old female child was brought to the hospital because of abnormal appearance and growth retardation. Antenatal history revealed full-term birth at 41 weeks of gestation. She was the second child (birth weight 1.9 kg) of a gravida 2, para 2 (G2P2) mother. There was no evidence of intrauterine fetal distress or hypoxia. The placenta was small and calcified, with oligohydramnios. Immediately after birth, the child was found to have a weak cry, cyanosis, and abnormal appearance, and had an Apgar score of 7 at 1 min and 9 at 5 min. The height (66 cm) and weight (6.5 kg) of the child were three percentage points below the reference height and weight of children of the same age. Her respiratory rate was 26 cycles/min and heart rate was 110 beats/min. The general condition appeared good; breath sounds were clear bilaterally, cardiac sounds were strongly audible, and a continuous murmur was heard at the second intercostal space towards the left margin of the sternum. Abdominal examination was unremarkable and did not reveal any palpable organomegaly. Neurological examination showed a soft neck, negative Babinski’s sign bilaterally, and normal muscle tension. Gesell developmental scale test showed a development quotient of 36, which corresponds to severe mental disability. Unusual findings pertaining to appearance included (Fig. 1): fair skin, small palpebral fissures, low-set ears and a preauricular skin tag on the left side, retrognathia, and uneven color distribution of the skin together with patchy pigmentation. Oral examination showed tooth fusion.

**Fig. 2:**
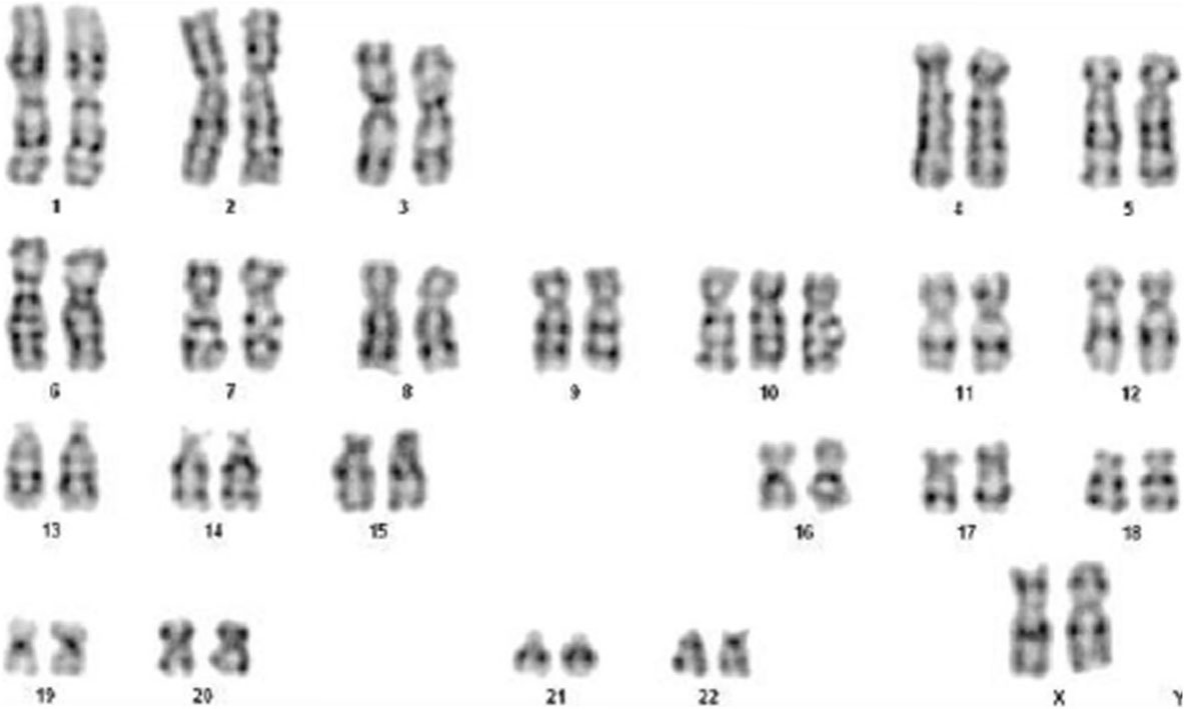
Karyotype analysis: analysis of 100 split phase, 42 mitotic phase, karyotype of 47, XX

**Fig. 3:**
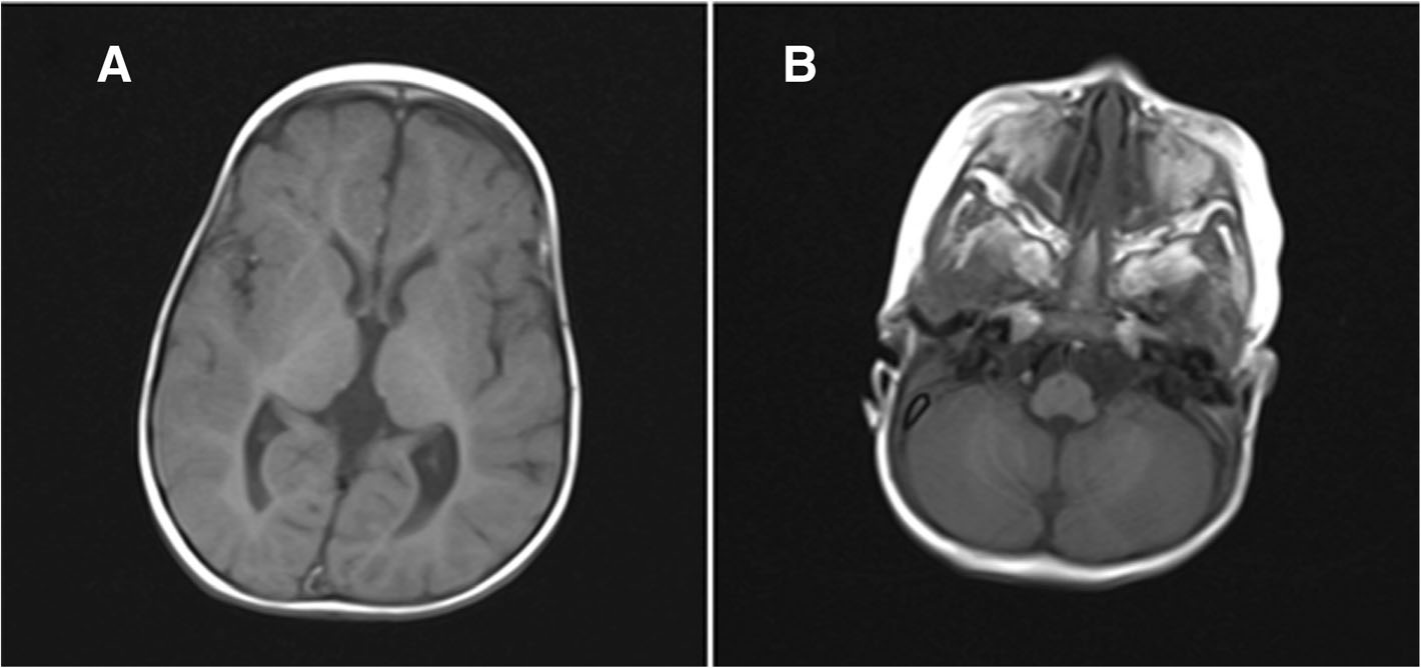
**a** The vermis of the cerebellum is smaller and is indicative of Dandy–Walker syndrome. **b** Widening of the ventricular and subarachnoid spaces

**Fig. 4:**
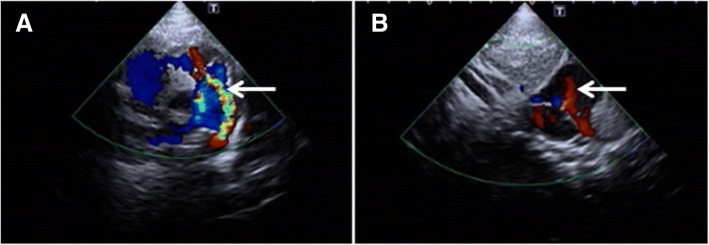
Echocardiography showed (**a**) a patent ductus arteriosus and (**b**) patent foramen ovale

Additional investigations revealed a standard chromosomal pattern; fluorescein in situ hybridization (FISH) analysis of blood revealed a female mosaicism karyotype 47, XX, + 10/ 46, XX with trisomy 10 in 42% of metaphases in the blood (Fig. 2). Cranial MRI showed (Fig. 3) bilateral widening of the frontal subarachnoid space, characteristic of the Dandy–Walker syndrome and enlarged ventricles. Echocardiography was conducted to investigate the abnormalities detected on cardiac auscultation (Fig. 4) and revealed the following: the descending aorta formed a multicolored blood-flow signal to the main pulmonary artery, with a flow width of 2.2 mm. The shunt persisted throughout the cardiac cycle. Doppler detected a maximal shunt velocity of 465 cm/s, with a mean pressure gradient of 86 mmHg. A 2-mm wide oblique separation was seen in the middle of the septum, which was indicative of patent ductus arteriosus (PDA) and a patent foramen ovale. No abnormalities were detected on amino acid and carnitine spectrum analysis for inherited metabolic diseases. Routine investigations of blood, urine, stool, liver function, renal function, myocardial enzymes, fasting blood sugar, and thyroid function were normal. The child’s mother and father were 34 and 36 years of age, respectively, at the time of her birth, whereas her elder sibling (brother) was 8 years old. The phenotype and chromosomal patterns in these close family members were normal. At the time of writing, the child has been followed up for 3 months after the diagnosis. At the most recent follow-up, her height has increased by 2 cm and weight has increased by 0.6 kg.

## Discussion and conclusions

The reported annual incidence of chromosomal abnormalities is approximately 100,000 in neonates in China; chromosomal abnormalities are detected in 0.3% of infants [3]. However, given the high morbidity and mortality attributable to chromosomal disorders, early diagnosis and intervention are important to improve the quality of life of the affected children. An abnormal chromosome number is the most commonly encountered chromosomal disorder in clinical settings. Trisomy of chromosomes 13, 18, and 21, as well as that of X and Y are the most common chromosomal abnormalities, and account for more than 95% of chromosomal aneuploidy. However, live-born carriers of trisomy 10 are rare [2, 4]. The other reported instances of trisomy 10 are part of the leukemic cell lines or found in tumor specimens. Trisomy 10 has the distinction of being the sole cytogenetic abnormality detected in acute myeloid leukemia, with the incidence ranging from 0.2 to 0.5% [5]; however, it has rarely been observed in acute lymphoblastic leukemia or other cancers [6–8].

The following salient features distinguish this case from the previously reported cases: (1) Apart from trisomy 13, 18, and 21, and that of the X and Y chromosomes, trisomy of other chromosomes is rarely detected in live-born children, as these typically result in early abortion unless occurring as a mosaicism; a live-birth with trisomy 10 is especially rare. In the present case, the general condition of the child was good, and she had survived for a relatively long period. Moreover, comprehensive clinical data were available. (2) Trisomy 10, in this case, presented with neurological deficits, in addition to abnormalities such as retrognathia, cardiac defects, growth retardation, and other characteristic phenotypic changes. This phenomenon was not observed in the previously reported cases of trisomy 10. (3) The earlier reports are relatively superficial and lack in-depth characterization of abnormalities, particularly the neurological abnormalities. To our knowledge, life expectancy of children with trisomy 10 has not been confirmed. This is likely attributable to the paucity of long-term follow-up data. In the cases reported till date, survival ranged from 37 days to 5 years and 4 months [1]. Given the child’s good general condition, there is scope for long-term follow-up. Furthermore, the chromosomal karyotype of her parents and brother are normal, which indicates that the trisomy 10 was caused by a genetic mutation. Given the rarity of trisomy 10, this case report is of much clinical value.

Trisomy 10 was first reported in 1973 [9]. Most of the subsequent reports were those of a mutation in some cells in some patients with leukemia who presented with trisomy 10 [6]. Other reports, largely based on prenatal diagnosis, identified fetuses with trisomy 10; however, most of these fetuses were aborted in early pregnancy [10]. To our knowledge, only 6 live-births with trisomy 10 have been reported in the literature, and most of these were shown to be mosaicism with an euploid cell line. The first documented case of trisomy 10 without euploid cells in a live-born was reported in 1997 [4]; however, the cell karyotype was 47, XX, + 10/45, X. The present case is one of mosaicism trisomy 10. The case report illuminates peculiar clinical characteristics and functional derangement associated with trisomy 10, which may facilitate a better understanding of the disease and possibly help in early diagnosis.

To the best of our knowledge, this is the first reported case of mosaicism trisomy 10 with the Dandy–Walker syndrome. The Dandy–Walker syndrome is a rare congenital neurological disorder characterized by occlusion of the median foramen, lateral foraminal atresia, and cerebellar dysplasia, which may cause psychomotor retardation and increased intracranial pressure. Previous reports have shown that the Dandy–Walker syndrome is often associated with deranged development of cerebellum and the surrounding structures during the early embryonal period. Some patients had chromosomal abnormalities involving chromosomes 3, 9, 13, 18, and 21; however, the exact mechanism is not fully clarified [11]. To our knowledge, this is the first reported case of trisomy 10 coexisting with the Dandy–Walker syndrome. The blood ammonia level of the present child was initially found to be slightly elevated. In order to rule out any inherited metabolic disease, we performed amino acid and carnitine spectrum analysis. However, the results were normal, and the blood ammonia level was spontaneously restored to normal. The significance of this case report is that it provides a comprehensive understanding of trisomy 10. Prenatal diagnosis, in such cases, could help minimize the economic and social burden imposed by this chromosomal aberration.
